# Two Cases of Impaired Wound Healing Among Patients With Major Head and Neck Free-Flap Reconstruction in the Setting of COVID-19 Infection

**DOI:** 10.7759/cureus.20088

**Published:** 2021-12-01

**Authors:** Daniel Inouye, Sheng Zhou, Bhavishya Clark, Mark Swanson, Tamara Chambers

**Affiliations:** 1 Otolaryngology-Head and Neck Surgery, Keck School of Medicine at University of Southern California, Los Angeles, USA

**Keywords:** head and neck surgery, covid-19, sars-cov-2, free tissue transfer, ­wound healing, microvascular reconstruction

## Abstract

Due to the microvascular effects of severe acute respiratory syndrome coronavirus 2 (SARS-CoV-2), head and neck reconstructive surgeries utilizing free tissue transfers may be profoundly affected by SARS-CoV-2 infection in the immediate postoperative period. Our objective is to describe two adult patients who developed SARS-CoV-2 after undergoing relatively uncomplicated segmental mandibulectomies. In both cases, the patients were initially negative for SARS-CoV-2, underwent relatively uncomplicated segmental mandibulectomies with fibula free flap reconstructions, and were later discharged in stable conditions. Both patients subsequently experienced significant infectious sequelae at the donor and recipient sites with near-total split-thickness skin graft loss in the donor sites in the setting of postoperative SARS-CoV-2 infection. The first patient developed sepsis and gangrenous changes to his fibula donor site requiring four operative debridements and partial amputation with subsequent osteomyelitis of the remaining fibula. The second patient experienced dehiscence of the oral fibula free flap as well as a 22 cm phlegmon at the fibula donor site that required surgical debridement. In consideration of these cases, SARS-CoV-2 infection during the immediate postoperative period of head and neck reconstruction procedures may elevate the risk of major wound complications. Special consideration must be taken when performing free tissue transfers during the COVID-19 pandemic.

## Introduction

Our clinical understanding of the coronavirus disease 2019 (COVID-19) pandemic is rapidly evolving. The causative virus known as severe acute respiratory syndrome coronavirus 2 (SARS-CoV-2) has been shown to cause endothelial cell inflammation and vasculitis with microvascular dysfunction and thrombosis [1]. Patients with SARS-CoV-2 have alterations of hemostatic parameters including thrombocytopenia, elevated D-dimer levels, and fibrinogen degradation products [2]. A post-mortem study of SARS-CoV-2 patients found microvascular thrombosis in all major organs with inflammatory cell infiltration, immune complex deposition, and viral inclusions on histology and electron microscopy [1]. To help explain these changes, several mechanisms of circulatory disruption have been postulated, including direct tissue tropism, extracellular histone cytotoxicity, complement complex deposition, alteration of pericytes, and inflammatory mediators causing endothelial cell damage [1,3].

The effects of SARS-CoV-2 on the microcirculatory system are of particular concern in reconstructive surgery. In otolaryngology, skin grafts, tissue rearrangements, and microsurgical tissue transfer procedures are frequently utilized to address head and neck tissue defects [4-5]. Success rates after head and neck free tissue transfer are typically greater than 95% [5]. These procedures require meticulous preparation of donor and recipient tissues with emphasis on maintaining healthy vasculature and creation of microsurgical anastomoses [4,6-8]. Skin grafts rely on a three-step process to establish vascularity for long-term success: imbibition, inosculation, and revascularization. In the first 24-48 hours (imbibition), graft capillaries receive nourishment from the underlying wound bed through simple diffusion, and the donor and recipient tissues become adhered via fibrin [9-11]. Over the next 24-36 hours (inosculation), new vasculature from the recipient site grows towards and interfaces with the donor tissue [9-11]. Ultimately, new vessels form to serve as collateral circulation (neovascularization) [9-11]. Neovascularization for free flaps can take at least four weeks for proper establishment, though this process can vary as it is dependent on multiple factors including the site of reconstruction, the quality of the recipient wound bed, and the type of flap utilized [12].

In consideration of these factors, SARS-CoV-2 infection during the perioperative period of head and neck reconstruction procedures may elevate the risk of major wound complications. Here, we present a case series of two patients profoundly affected by the deleterious effects of SARS-CoV-2 in the immediate postoperative period after free tissue transfer at a tertiary academic medical center from June to July 2020. Of note, these two cases were performed prior to SARS-CoV-2 vaccinations being publically available.

## Case presentation

Case report 1

The first patient was a 55-year-old man who presented with a pathologic mandible fracture. He was subsequently found to have a soft tissue mass in his gingivobuccal sulcus extending to his retromolar trigone with erosion extending from the mid-body of his left mandible to the ramus on computerized tomography. The patient also had significant ipsilateral lymphadenopathy involving neck levels 1B through 3 with the largest node measuring 21 mm. Biopsy of the erosive mass was positive for squamous cell carcinoma (SCC). The patient is a former smoker (an approximately 40 pack-year history) with a history of type 2 diabetes mellitus and hypertension. He had an appropriate body mass index (BMI) of 27.29 kg/m^2^ but displayed mildly low albumin levels of 3.0 g/dL indicating possible malnutrition. Three days prior to surgery, he tested negative for SARS-CoV-2 on a nasopharyngeal swab using PCR. The patient underwent a left segmental mandibulectomy with modified left neck dissection in addition to tracheostomy and gastrostomy tube placement. His mandible defect was reconstructed with a right-sided fibula free flap. A split-thickness skin graft (STSG) from his left lateral thigh was used to close the free flap donor site. There were no intraoperative complications. Final pathology revealed a T4aN3bM0 SCC with multiple high-grade features, including positive extranodal extension, perineural invasion, and lymphovascular invasion. The patient’s postoperative course was complicated by a 1 cm area of dehiscence in the neck on postoperative day (POD) 6, which was managed with local wound care. On POD7, he was decannulated from his tracheostomy and the cast over the fibula site was removed. There was complete STSG adherence without loss of any portion of the graft. He was discharged the same day. At the time of his discharge, he displayed no COVID symptoms. At his first follow-up on POD13, the area of dehiscence in the neck was noted to be improving though there was a minor retraction of the STSG from the edges of the fibula donor site. On POD20, the patient presented to the emergency room with complete STSG dehiscence and dry, gangrenous changes of the fibula free flap donor site. He was newly SARS-CoV-2 positive on repeat testing via the Simplexa COVID-19 Direct RT-PCR assay (DiaSorin Molecular, Cypress, CA) using a nasopharyngeal swab but was asymptomatic. Blood tests and chemistries were notable for leukocytosis though he had no systemic infectious symptoms. The patient was afebrile with stable vital signs.

The patient was started on ceftriaxone and vancomycin and received four operative debridements with V.A.C. VeraFlo wound vacuum placement (KCI, Acelity, San Antonio, TX). Initially, the necrotic wound tracked down to the lateral malleolus and ankle. Areas of purulence surrounded his gastrocnemius muscles. Tissue cultures showed *Klebsiella aerogenes*, *Enterobacter cloacae*, and *Peptostreptococcus*. Bone biopsies were also positive for *Klebsiella* and *E. cloacae*. He remained hospitalized under the careful co-management of infectious disease specialists. No additional medications were given for SARS-CoV-2, as the patient did not demonstrate any respiratory sequelae at that time.

On POD40, the patient redeveloped left-sided cervical lymphadenopathy and another left-sided mandible mass. Needle biopsy confirmed the diagnosis of recurrent SCC. Repeat head and neck imaging showed extensive metastatic lymphadenopathy in the bilateral submental and submandibular space in addition to a large heterogeneously enhancing mass in the left mandible measuring up to 5.7 cm. Due to the aggressive nature of his cancer, immediate initiation of chemotherapy and radiation was recommended. His prior fibula donor site did not respond well to operative debridement, systemic antibiotics, and local wound care with the VeraFlo wound vacuum. On POD51, the patient underwent a partial amputation of his right lower extremity and an awake tracheostomy. He was discharged home soon after with a plan for immediate outpatient adjuvant therapy.

Before being able to initiate his treatments, the patient was readmitted on POD56 with signs and symptoms of sepsis. Admission labs showed leukocytosis of 43.7K white blood cells and lactate of 4.4 mmol/L. He was hypotensive, febrile, and tachypenic. Lower extremity scans revealed osteomyelitis of the remaining fibula with a new soft tissue abscess. Blood cultures had no positive growth. Repeat head and neck imaging showed dramatic progression in the patient’s cervical lymphadenopathy and left mandible mass, which had nearly tripled in size (15.3 cm from 5.7 cm on POD40). The tumor was non-resectable and too large for radiation. The patient elected against chemotherapy and was discharged to home hospice on POD63.

Case report 2

The second patient is a 64-year-old gentleman who initially presented with pT2N1MX (Stage III) SCC of the oral tongue in 2017. He underwent a partial glossectomy, right modified radical neck dissection, and tracheostomy. The tongue was reconstructed with a left radial forearm free flap with lateral left thigh STSG coverage of the arm defect. His postoperative course was uncomplicated, and he completed adjuvant radiation in 2018. In March 2020, he had local recurrence at the interface of the native tongue and radial forearm free flap. At this time, left partial glossectomy, marginal mandibulectomy, left modified radical neck dissection, and repeat tracheostomy were performed. The oral defect was repaired with a right radial forearm free flap with an ipsilateral thigh STSG. The patient’s perioperative course was again uncomplicated without wound breakdown. Given his clear margins of resection, negative lymph nodes, and prior history of radiation treatment, no adjuvant therapy was pursued at that time. In August 2020, a second recurrence of his cancer occurred with the involvement of the mandible. There was no pathologic cervical lymphadenopathy. The patient was a former smoker (an approximately 25 pack-year history) who quit in 2018. The patient had no other past medical history and was reasonably well-nourished with albumin of 4.3 g/dL and a BMI of 23.2 kg/m^2^. He received a segmental mandibulectomy with left fibula free flap reconstruction in September 2020. Three days prior to surgery, he had a negative nasopharyngeal swab test for SARS-CoV-2. After the resection, the patient was staged as recurrent T4aN0. Following the surgery, the patient’s postoperative course was complicated by a small area of dehiscence along the central neck incision, which was managed with local wound care. On POD6, the cast over the fibula site was removed and the STSG was adherent without loss of any portion of the graft. On POD9, the patient was seen in the clinic with all surgical sites in the appropriate stages of healing.

On POD16, the patient presented to the emergency department with a two-day history of chills and fevers with increased pain at the fibula donor site. Admission vitals and labs were unremarkable. Upon presentation to the emergency room, the patient was noted to have partial loss of the skin graft on his left leg with surrounding erythema. Though the intra-oral fibula flap was viable, the sutures holding it in place were dehiscent and plating hardware was exposed. He tested positive for SARS-CoV-2 through the Simplexa COVID-19 Direct RT-PCR assay nasopharyngeal swab.

The patient was admitted for intravenous ciprofloxacin based on early lower extremity cultures. The area of intraoral dehiscence was explored and managed with local wound care. Lower extremity imaging was positive for a 22 cm phlegmon. He was taken to the operating room for wide local debridement and wound washout. Purulence was tracking to the posterior calf along previously dissected surgical planes. The non-viable STSG was gently debrided. The VeraFlo wound vacuum was placed. The wound cultures taken in the operating room showed polymicrobial growth with *Enterococcus faecalis*, *Enterobacteriaceae*, methicillin-susceptible *Staphylococcus aureus*, and *Streptococcus agalactiae*. Antibiotic coverage was expanded to include vancomycin, cefepime, and metronidazole. The VeraFlo wound vacuum was exchanged for a traditional model five days after placement. A healthy wound bed was seen with new granulation tissue. The patient was discharged on POD30 with the wound vacuum in place, daily oral wound care, and a plan for long-term antibiotics.

## Discussion

Microsurgical flap procedures in head and neck surgery rely on the establishment of proper revascularization for success and include multiple wound sites. Combining this with the predilection of SARS-CoV-2 to cause microvascular pathology suggests that free-flap reconstructions may face unique challenges with concurrent SARS-CoV-2 infection. Several case studies have described remarkable surgical site complications in the setting of SARS-CoV-2 infection [13-15]. Benmoussa et al. described the failure of a chimeric thoracodorsal artery perforator flap and a free fibula flap performed for gingival mandibular squamous cell carcinoma, and da Silveira et al. describe significant sternal dehiscence following a coronary artery bypass graft [14-15]. In these previously published cases, the procedures were performed early in the COVID-19 pandemic prior to preoperative screening. However, these studies are limited by the unknown timeline for SARS-CoV-2 infection. One such study by Talmor et al. described a case of pedicled nasoseptal flap necrosis and failure with a positive postoperative SARS-CoV-2 test [13]. To our knowledge, we are the first to describe near-total STSG loss with severe infectious sequelae in both the donor and recipient sites in major free tissue transfer in the setting of postoperative SARS-CoV-2 infection for patients with negative preoperative testing.

The microcirculatory effects of SARS-CoV-2, as well as the necessity of proper neovascularization, are particularly critical factors in the two cases of free tissue transfers presented here. Both patients tested negative for SARS-CoV-2 three days prior to surgery, underwent uncomplicated fibula free-flap reconstructions after segmental mandibulectomies, and were discharged home in stable conditions. These patients subsequently developed significant donor site infections and skin graft loss associated with SARS-CoV-2 infection during the recovery period, and the degree of flap dehiscence and donor site infection was severe in both cases. The first patient also developed severe sepsis and required a partial lower extremity amputation. However, the patient was at a higher risk of wound complications due to his medical comorbidities and initial nutritional status.

In the second case, although found to be well-healing for two weeks after surgery, dehiscence of the oral fibula free flap occurred suddenly 16 days after surgery. This is an uncommon timeline for microvascular tissue transfer failures [8]. Notably, this patient had previously received two prior tissue transfer surgeries without any wound complications, even after radiation. Unlike the first patient, this patient did not have any new medical diagnoses or indications of significant malnutrition preoperatively. However, his most recent operation was unique due to postoperative SARS-CoV-2 infection.

Grafts and free tissue transfers are integrated through imbibition, inosculation, and revascularization. At one week after surgery, neither of these patients showed signs of imminent donor or recipient site morbidities. The initial stages of imbibition and inosculation were completed during the first week when they were hospitalized and not in contact with SARS-CoV-2. Neovascularization was likely impeded by infection with SARS-CoV-2 approximately two weeks after surgery in both these cases.

At the time of this publication, research is beginning to illuminate how SARS-CoV-2 mediates microvascular changes through the production of angiotensin II (Ang II). The angiotensin-converting enzyme 2 (ACE2) is the functional receptor of SARS-CoV-2 [16-17]. ACE2 is highly expressed on endothelial cells within all tissues, including the nasopharynx, oropharynx, and lungs [1,18]. ACE2 is also expressed on arterial smooth muscle cells, as well as the pericytes of the microcirculatory system that hold a critical role in vascular homeostasis and inflammation [1,18]. The binding of SARS-CoV-2 to ACE2 additionally leads to elevated levels of Ang II [19]. Besides being a potent vasoconstrictor, Ang II is also a powerful pro-inflammatory molecule that increases free radical generation causing oxidative stress, mitochondrial dysfunction, endothelial cell damage, and thrombosis [19]. Zoufaly et al. recently described the first case of severe SARS-CoV-2 infection treated with human recombinant soluble ACE2 (hrsACE2) and observed marked reductions in Ang II, as well as reductions in inflammatory cytokines associated with the pathophysiology of COVID-19 including interleukin 6 and interleukin 8 [20]. These findings suggest an important role for ACE2 and Ang II in the pathogenesis of major wound complications associated with SARS-CoV-2 infection. Further research is needed to better understand the mechanism behind circulatory disruptions caused by SARS-CoV-2. While the first patient had comorbidities that may have also contributed to his wound complications, the second patient's disease course more strongly suggests that SARS-CoV-2 has a detrimental role in wound healing. Future directions include the study of ACE inhibitors and hrsACE2 to mitigate or prevent SARS-CoV-2 complications.

## Conclusions

Special consideration must be taken when performing tissue transfer procedures during the COVID-19 pandemic. Providers should utilize preventative measures to reduce the risk of SARS-CoV-2 infection in the perioperative period. Patients and their families should also be educated on the possible deleterious side-effects of SARS-CoV-2 on flap and wound healing, and emphasis should be placed on preventing at-home exposure to SARS-CoV-2 after discharge. Additionally, patients at home should be isolated from other members of the household when possible, and screening family members for SARS-CoV-2 should be considered. We recommend more frequent wound examinations and early detection through careful monitoring for the development of symptoms associated with SARS-CoV-2 infection during all patient interactions.
